# Prognostic analysis of gastric signet ring cell carcinoma and mucinous carcinoma: a propensity score-matched study and competing risk analysis

**DOI:** 10.18632/aging.104048

**Published:** 2020-10-31

**Authors:** Chao-Tao Tang, Youxiang Chen, Chunyan Zeng

**Affiliations:** 1 Department of Gastroenterology, The First Affiliated Hospital of Nanchang University, Nanchang, China

**Keywords:** SEER, signet ring cell carcinoma, mucinous gastric cancer, competing risk regression model, propensity score matching

## Abstract

Background: Limited evidence and contradictory results have been reported regarding the impact of signet ring cell carcinoma (SRC) and mucinous gastric cancer (MGC) classifications on the prognosis of gastric cancer (GC).

Results: Information on 6017 patients and 266 patients was extracted from the SEER database and our hospital records, respectively. We found that patients with MGC had a better survival rate than those with SRC (P=0.012), but in the early stage, MGC was a risk factor for a poor prognosis. After PSM, for both patients from the SEER database and our hospital, the prognosis of patients with SRC was poorer than that of patients with MGC (P<0.05), but patients with MGC in early-stage GC showed poorer survival. Additionally, SRC was demonstrated to be a risk factor in the multivariate competing risk regression model for cancer-specific survival.

Conclusion: Patients with SRC may have a worse prognosis than those with MGC, but for early-stage GC, patients with SRC have a better prognosis than those with MGC.

Method: Patients from the SEER database and from our hospital diagnosed with SRC or MGC were included in a Cox regression analysis, multivariate competing risk model and propensity score matching (PSM) analysis.

## INTRODUCTION

Gastric cancer (GC) is one of the most common types of cancer worldwide, and there were over 1,000,000 new cases of GC and an estimated 783,000 related deaths in 2018; GC is especially common in East Asia, such as Japan and Korea, compared with North America and Europe [1]. According to the WHO classification [2], GC is divided into four main types, including signet ring cell carcinoma (SRC) and mucinous gastric carcinoma (MGC). MGC accounts for a smaller proportion of GC cases than SRC and is defined as a tumor containing more than 50% extracellular mucin, while SRC is defined as a tumor with intracellular mucin pools causing the nucleus to be squeezed to the margin of the cell [2]. With respect to the prognosis of these two types of cancer, some small population-based studies have been performed. Although MGC is rare, it is usually detected at an advanced stage, leading to a poorer prognosis than common gastric adenocarcinoma. According to several studies, the 5-year survival rate is 30%-50%, which is obviously lower than that of patients without MGC [3, 4]. Compared with MGC, SRC has been regarded as an independent predictive factor of survival [4]. As described in the latest large-population study [5], the 5-year survival rate was 46.1%, while other studies reported a 5-year survival rate of 30%-40% [6, 7]. To date, several studies have compared the differences in survival between MGC and SRC. Some studies have reported patients with MGC to have better survival than those with SRC [8–11], while a few studies have shown no significant difference in survival between the two types of cancer [12, 13]. Furthermore, some studies reported that patients with MGC had a poorer prognosis than patients with other histological types of GC [14–16]. Considering the contradictory results, some statistical reasons for these differences should be proposed. First, the small sample sizes and different populations used to investigate the differences are major causes of the inconsistencies; additionally, incomplete statistical analyses should not be overlooked. Finally, differences in race are also an important factor and should be considered. Therefore, we conducted a comprehensive analysis to compare the survival rate of MGC and SRC patients via the most detailed statistical methods.

In our study, we extracted information for 6017 patients from the SEER database and 266 patients from our hospital to investigate the survival difference between MGC and SRC. Cancer-specific survival (CSS) and overall survival (OS) were regarded as the observation indices to evaluate the prognosis in the two groups. We describe the clinicopathological characteristics based on a large-population analysis and increase the knowledge of these two types of tumors with the use of propensity score matching (PSM) and competing risk analysis.

## RESULTS

### Basic clinical information of the patients

As shown in the flow chart, we finally extracted data for 6017 patients diagnosed with SRC or MGC between 2004 and 2015 from the SEER database, including 752 patients with MGC and 5265 patients with SRC (Supplementary Figure 1). Additionally, according to the inclusion and exclusion criteria (Supplementary Figure 2), we extracted data for 266 patients diagnosed with SRC or MGC between 2014 and 2020 from our hospital records. Then, according to the histological type of GC (SRC or MGC), we recorded the patient demographics, which are shown in Tables 1 and 2. As shown in Table 1, the incidence of early-onset GC in the SEER database was similar for SRC and MGC (10.54% vs 9.57%, P=0.416), while in our data, we found that early-onset GC was more frequent in SRC patients than in MGC patients (18.02% vs 5.16%, P=0.0008) (Table 2). The proportion of male patients with MGC was higher than that with SRC (69.41% vs 53.37%, P<0.001) (Table 1), which is in line with the results of our data (Table 2). In addition, MGC occurred in the cardia more often than SRC (34.57% vs 18.82%), and the size of MGC tumors was larger at diagnosis than the size of SRC tumors (>3 cm, 76.19% vs 63.84%) (Table 1), which is consistent with our data (Table 2). The incidence of early-stage MGC was lower than that of early-stage SRC (13.56% vs 22.45%, P<0.05), which is also in agreement with our data (25.82% vs 31.53%). Although our data show that patients with MGC tended to have LNM (81.29% vs 53.15%, P<0.001) (Table 2), there was no significant difference in LNM or distant metastasis between SRC and MGC (P>0.05) (Table 1).

**Table 1:** Basic characteristics of patients at diagnosis.

**Variables**	**Total (%)**	**Signet ring cell carcinoma**	**Mucinous Adenocarcinoma**	**P Value**
n	6017	5265(87.50%)	752(12.5%)	
Age				0.416
<=45	627(10.42%)	555(10.54%)	72(9.57%)	
>45	5390(89.58%)	4710(89.46%)	680(90.43%)	
Race				0.01
White	4140(68.81%)	3609(68.55%)	531(70.61%)	
Black	763(12.68%)	654(12.42%)	109(14.49%)	
Other	1114(18.51%)	1002(19.03%)	112(14.89%)	
Sex				0.000
Male	3332(55.38%)	2810(53.37%)	522(69.41%)	
Female	2685(44.62%)	2455(46.63%)	230(30.59%)	
Lymph node Metastasis				0.644
N0	2286(37.99%)	1988(37.76%)	298(39.63%)	
N1	2118(35.2%)	1857(35.27%)	261(34.71%)	
N2	1063(17.67%)	931(17.68%)	132(17.55%)	
N3	550(9.14%)	489(9.29%)	61(8.11%)	
Metastasis				0.054
No	4803(79.82%)	4183(79.45%)	620(82.45%)	
Yes	1214(20.18%)	1082(20.55%)	132(17.55%)	
Localization				0.000
Cardia	1251(20.79%)	991(18.82%)	260(34.57%)	
Fundus	173(2.88%)	148(2.81%)	25(3.32%)	
Body	645(10.72%)	582(11.05%)	63(8.38%)	
Anturm	1607(26.71%)	1413(26.84%)	194(25.80%)	
Pylorus	240(3.99%)	221(4.20%)	19(2.53%)	
Lesser curvature	724(12.03%)	662(12.57%)	62(8.24%)	
Greater curvature	334(5.55%)	310(5.89%)	24(3.19%)	
Overlappping/NOS	1043(17.33%)	938(17.82%)	105(13.96%)	
Size				0.000
≤2cm	1163(19.33%)	1082(20.55%)	81(10.77%)	
≤3cm	920(15.29%)	822(15.61%)	98(13.03%)	
≤5cm	1534(25.49%)	1315(24.98%)	219(29.12%)	
>5cm	2400(39.89%)	2046(38.86%)	354(47.07%)	
Examined LNs				0.096
≤16	3853(64.04%)	3351(63.65%)	502(66.76%)	
>16	2164(35.96%)	1914(36.35%)	250(33.24%)	
Historic Stage A				0.611
Localized	1674(27.82%)	1469(27.90%)	205(27.26%)	
Regional	2862(47.57%)	2492(47.33%)	370(49.20%)	
Distant	1481(24.61%)	1304(24.77%)	177(23.54%)	
T Stage				0.000
Tis/T1	1284(21.34%)	1182(22.45%)	102(13.56%)	
T2	2446(40.65%)	2077(39.45%)	369(49.07%)	
T3	1550(25.76%)	1351(25.66%)	199(26.46%)	
T4	737(12.25%)	655(12.44%)	82(10.90%)	

**Table 2:** Basic characteristics of patients at diagnosis from the First Affiliated Hospital of Nanchang University.

**Variables**	**Total (%)**	**Signet ring cell carcinoma**	**Mucinous Adenocarcinoma**	**P Value**
n	266	111	155	
Age				0.0008
<=45	28 (10.53%)	20 (18.02%)	8 (5.16%)	
>45	238 (89.47%)	91 (81.98%)	147 (94.84%)	
Sex				0.000
Male	182 (68.42%)	58 (52.25%)	124 (80%)	
Female	84 (31.58%)	53 (47.75%)	31 (20%)	
Lymph node Metastasis				0.000
N0	81 (30.45%)	52 (46.85%)	29 (18.71%)	
N1	48 (18.05%)	18 (16.22%)	30 (19.35%)	
N2	52 (19.55%)	16 (14.41%)	36 (23.22%)	
N3	85 (31.95%)	25 (22.52%)	60 (38.71%)	
Metastasis				0.296
No	233 (87.59%)	100 (90.1%)	133 (85.81%)	
Yes	33 (12.41%)	11 (9.9%)	22 (14.19%)	
T Stage				0.329
T1	75 (28.19%)	35 (31.53%)	40 (25.81%)	
T2	35 (13.16%)	18 (16.22%)	17 (10.97%)	
T3	27 (10.15%)	10 (9%)	17 (10.97%)	
T4	129 (48.5%)	48 (43.24%)	81 (52.26%)	
Localization				0.761
Cardia	9 (3.38%)	3 (2.71%)	6 (3.87%)	
Fundus	12 (4.51%)	4 (3.61%)	8 (5.16%)	
Body	65 (24.44%)	24 (21.62%)	41 (26.45%)	
Anturm	156 (58.65%)	70 (63.06%)	86 (55.48%)	
Overlappping/NOS	24 (9.02%)	10 (9%)	14 (9.03%)	
Size				0.000
≤2cm	48 (18.05%)	36 (32.43%)	12 (7.74%)	
≤3cm	48 (18.05%)	29 (26.13%)	19 (12.26%)	
≤5cm	91 (34.2%)	36 (32.44%)	55 (35.48%)	
>5cm	79 (29.7%)	10 (9%)	69 (44.52%)	
Examined_LNs				0.608
≤16	35 (13.16%)	16 (14.41%)	19 (12.26%)	
>16	231 (86.84%)	95 (85.59%)	136 (87.74%)	
Treatment methods				0.855
Conditional surgery	17 (6.39%)	6 (5.4%)	11 (7.1%)	
Laparoscopic surgery	225 (84.59%)	95 (85.6%)	130 (83.87%)	
Robotic surgery	24 (9.02%)	10 (9%)	14 (9.03%)	
Chemotherapy after surgery				0.721
No	162 (60.9%)	69 (62.16%)	93 (60%)	
Yes	104 (39.1%)	42 (37.84%)	62 (40%)	
Lymphatic vessel infiltration				0.0036
No	136 (51.13%)	71 (63.96%)	65 (41.94%)	
Yes	130 (48.87%)	40 (36.04%)	90 (58.06%)	

### Survival differences between SRC and MGC

**Figure 1:**
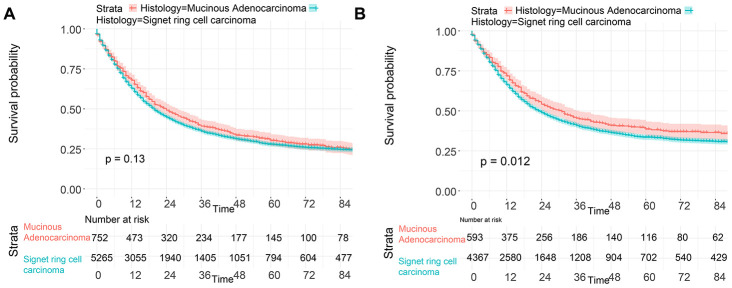
**Survival of GC patients with SRC and MGC.** (**A**) OS of SRC and MGC. (**B**) CSS of SRC and MGC.

**Figure 2:**
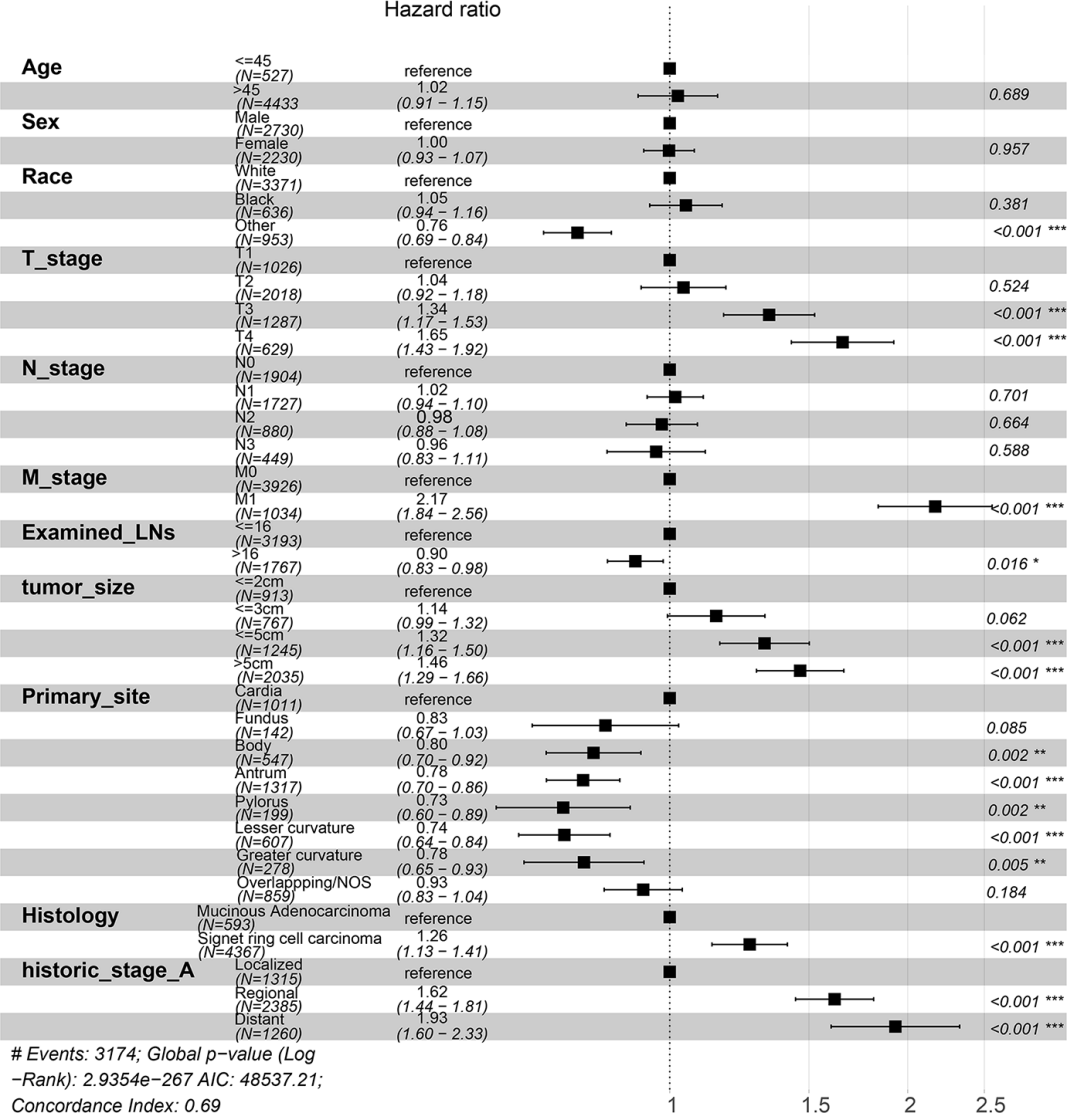
**Forest plot showing the results of the multivariate Cox regression model for exploring the potential risk factors for CSS in patients from the SEER database.**

**Figure 3:**
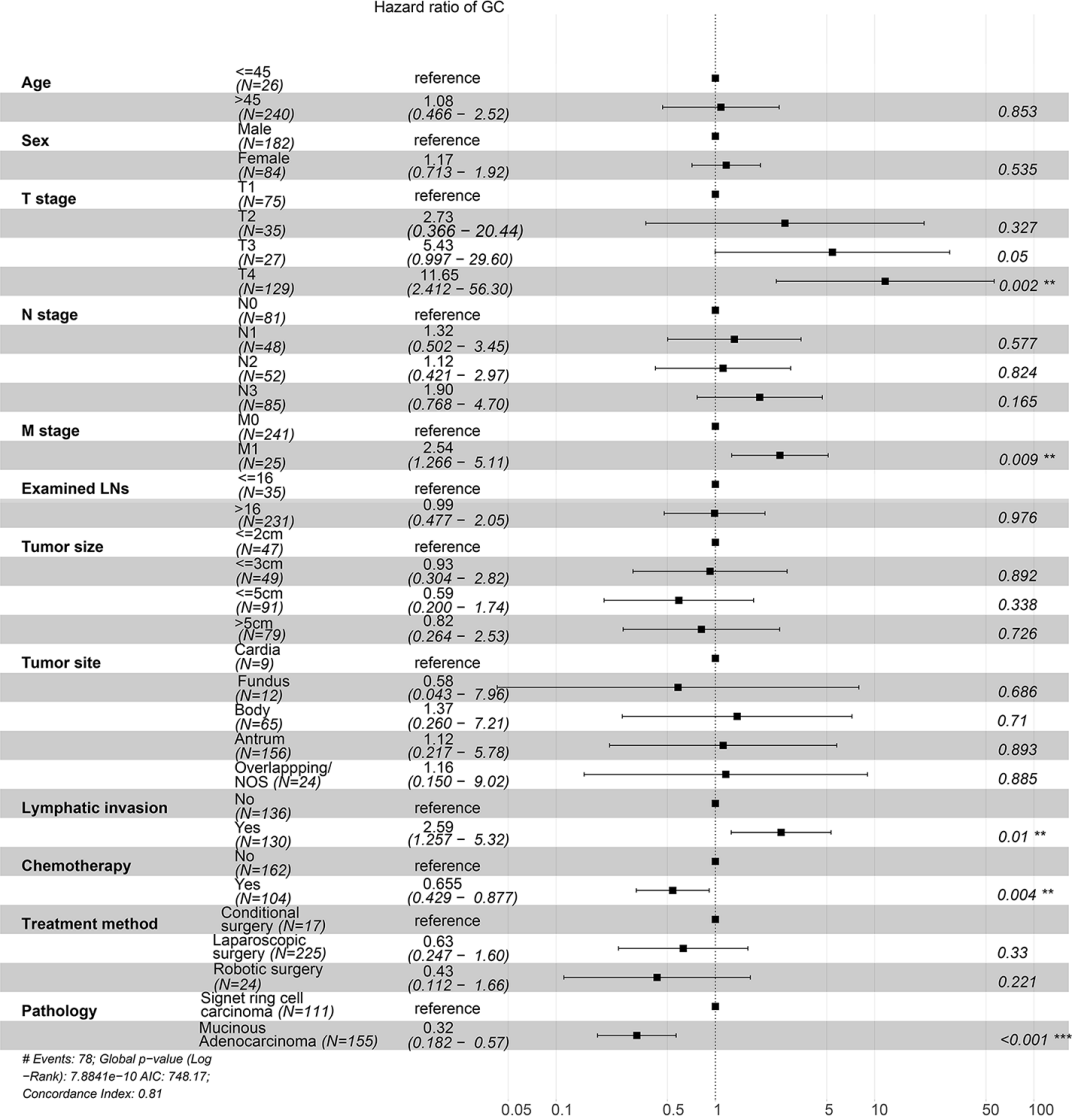
**Forest plot showing the results of the multivariate Cox regression model for exploring the potential risk factors for OS in patients from the First Affiliated Hospital of Nanchang University.**

**Figure 4:**
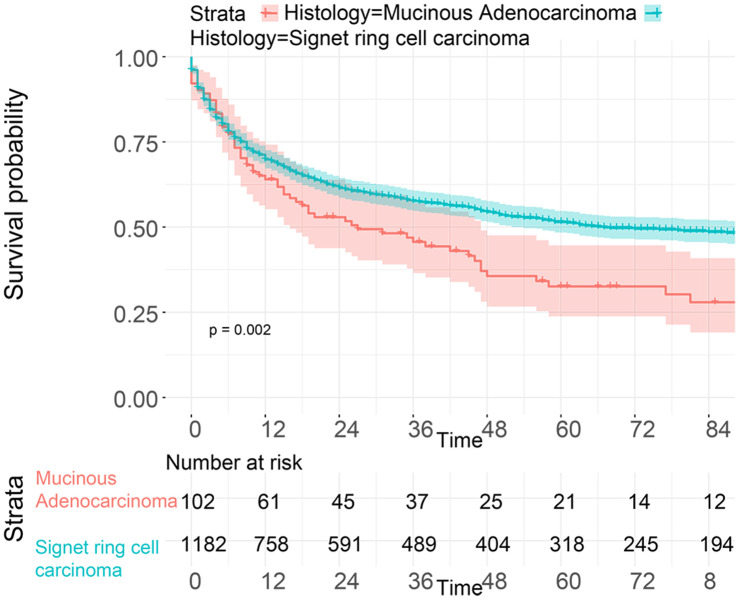
**OS analysis of patients from the SEER database with SRC and MGC in the early stage.**

**Figure 5:**
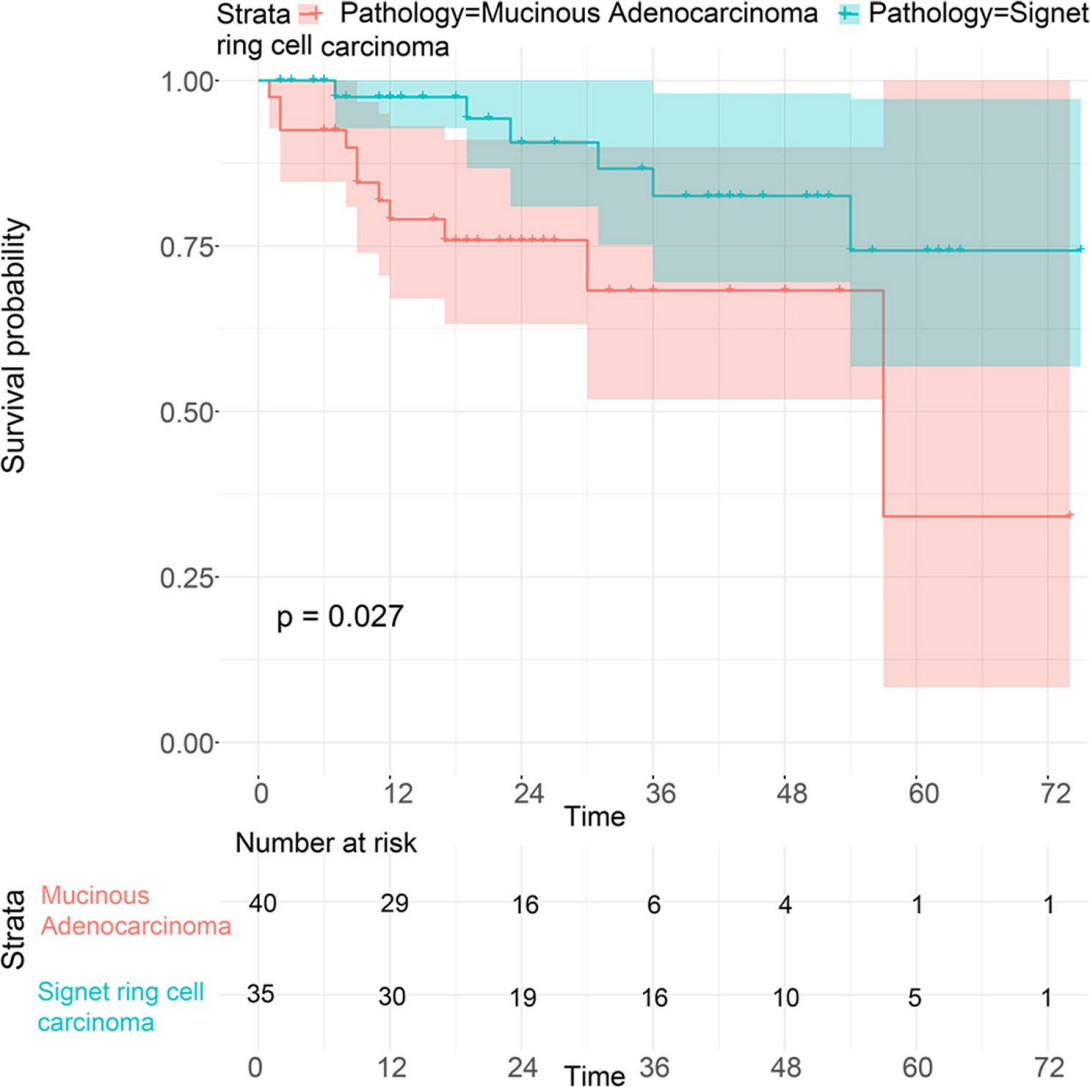
**Survival analysis of patients with SRC and MGC in the early stage from the First Affiliated Hospital of Nanchang University.**

To investigate the survival differences between SRC and MGC, we created a K-M survival curve. The 1-year, 3-year and 5-year OS rates of SRC were 63%, 37.5% and 27.5%, respectively, while those of MGC were 67.5%, 39.7% and 29%, respectively, with no significant differences (Figure 1A). However, in terms of CSS, the 1-year, 3-year and 5-year survival rates of MGC were better than those of SRC (P=0.012) (Figure 1B). In line with the results of the SEER database, our data revealed no significant difference in OS between the two histological types (P=0.77) (Supplementary Figure 3). We performed univariate and multivariate Cox regression analyses to determine the independent risk factors. As shown in Figures 2 and 3, both analyses indicated that distant metastasis, advanced tumor stage (T4) and SRC were independent risk factors for the survival of patients with SRC or MGC. Moreover, the results of the SEER database suggested that the number of examined LNs was a protective factor and that tumors located in the cardia predicted a worse prognosis (Figure 2). Based on our data (Figure 3), we found that lymphatic invasion was an independent risk factor, while chemotherapy was beneficial for patients. Interestingly, in the competing risk model, in both the univariate analysis and the multivariate analysis, we found that SRC had a higher rate of GC-related death than MGC (HR=1.329, 95% CI, 1.12-1.783, P<0.05) (Supplementary Figure 4 and Table 3). Moreover, in addition to age, we found that advanced T stage, LNM, large tumor size and distant metastasis were risk factors for patient survival (Table 3). Furthermore, we extracted data for patients with early-stage GC to explore survival differences between SRC and MGC. Inconsistent with the results of the previous analysis, we found that patients with MGC histology in the early stage had poorer survival than those with SRC histology (Figures 4 and 5). The 5-year survival rate of SRC was 54.5%, while that of MGC was only 34.2% (P=0.002).

**Table 3:** The result of competing risks regression with all possible risk factors in patients with gastric cancer.

**Variables**	**Subdistribution hazard ratio**	**P Value**
Age		**0.54**
<=45	Reference	-
>45	1.12(0.916-1.59)	0.409
Race		0.0009
White	Reference	-
Black	1.210(1.109-1.432)	0.023
Other	0.735(0.665-0.812)	0.000
Sex		0.8
Male	Reference	-
Female	0.990(0.926-1.057)	0.8
Lymph node Metastasis		
N0	Reference	-
N1	1.17(0.942-1.215)	0.052
N2	2.35(1.753-3.765)	0.005
N3	3.591(2.702-4.597)	0.000
Metastasis		
No	Reference	-
Yes	1.342(1.101-1.945)	0.000
Histology		0.000
Mucinous adenocarcinoma	Reference	-
Signet ring cell carcinoma	1.329(1.12-1.783)	0.000
Localization		0.65
Cardia	Reference	-
Fundus	0.909(0.860-1.201)	0.353
Body	0.934(0.857-1.125)	0.214
Anturm	0.921(0.887-1.127)	0.407
Pylorus	1.081(0.821-1.233)	0.532
Lesser curvature	0.874(0.739-1.029)	0.045
Greater curvature	0.943(0.812-1.138)	0.239
Overlappping/NOS	1.032(0.91-1.245)	0.25
Tumor size		0.000
≤2cm	Reference	-
≤3cm	1.13(1.019-1.405)	0.024
≤5cm	1.291.193-1.393)	0.000
>5cm	1.455(1.32-1.64)	0.000
Regional_nodes_examined		0.000
<=16	Reference	-
>16	0.659(0.526-0.745)	0.000
Historic Stage A		0.000
Localized	Reference	-
Regional	1.211(1.031-1.418)	0.000
Distant	1.585(1.239-1.943)	0.000
T Stage		0.000
T1	Reference	-
T2	1.193(1.072-1.43)	0.001
T3	1.214(1.05-1.317)	0.000
T4	1.539(1.412-2.01)	0.000

### Comparison of survival between SRC and MGC after matching

**Figure 6:**
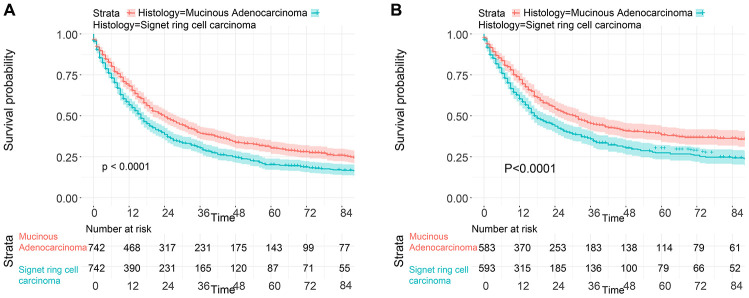
**Survival of GC patients from the SEER database with SRC and MGC after PSM.** (**A**) OS of SRC and MGC patients. (**B**) CSS of SRC and MGC patients.

**Figure 7:**
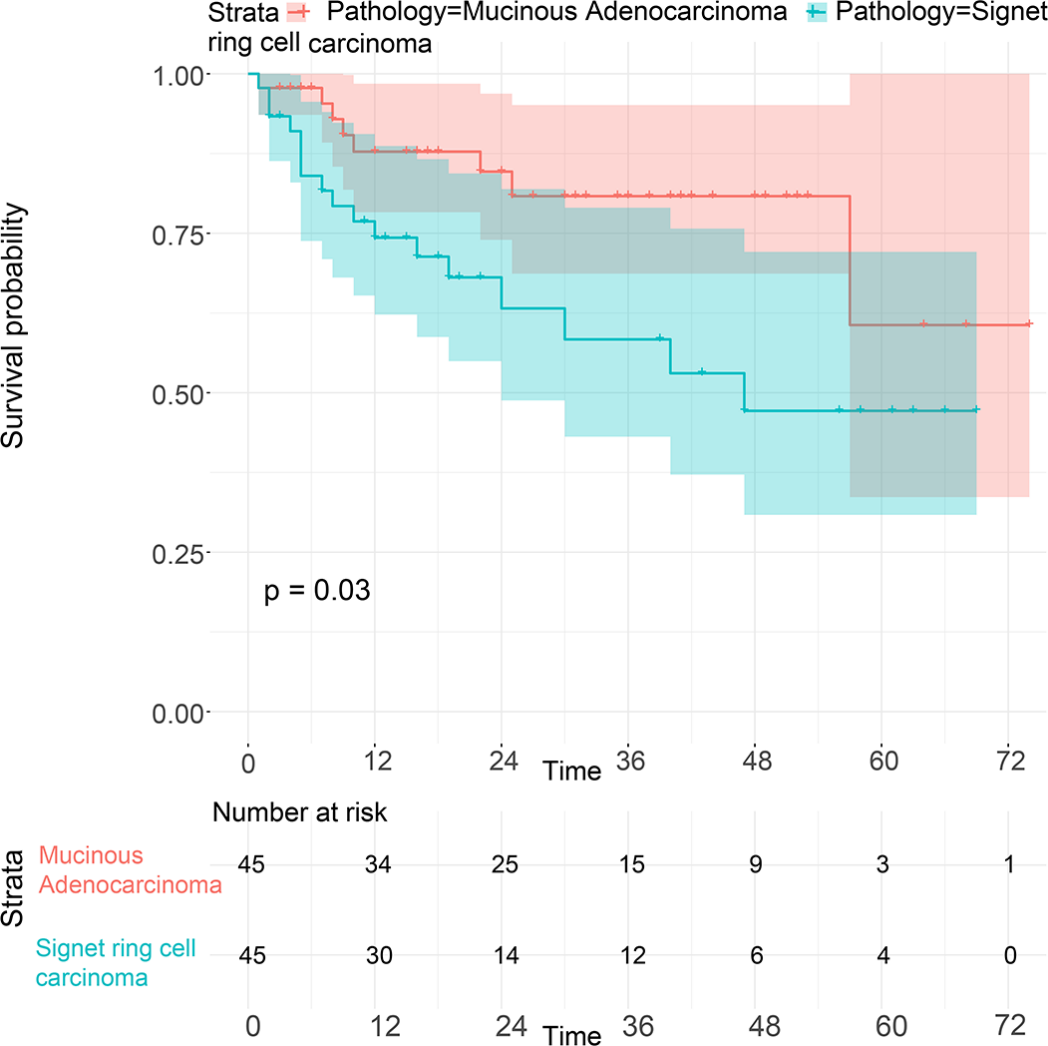
**Survival of GC patients with SRC and MGC from our hospital after PSM.**

**Figure 8:**
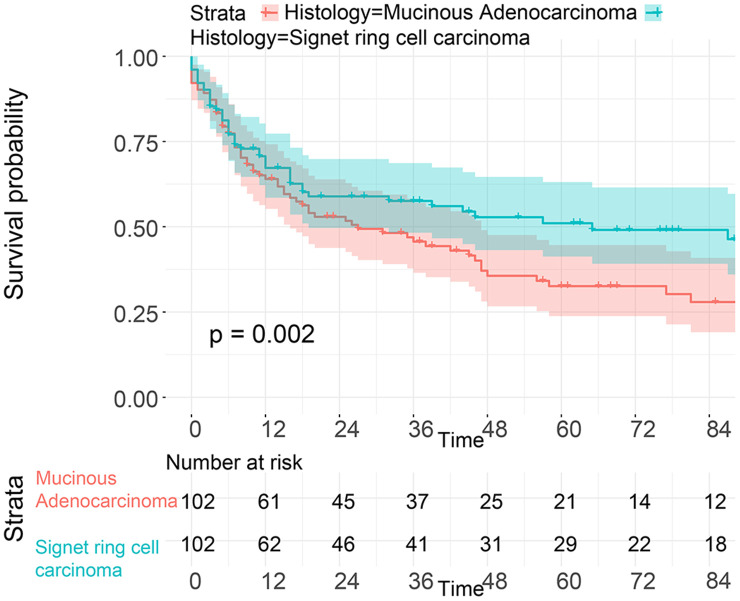
**Survival analysis of patients from the SEER database with SRC and MGC in the early stage after PSM.**

To balance the confounding factors, we performed PSM at a 1:1 ratio. As shown in Table 4, we matched 742 patients with SRC with 742 patients with MGC. The differences between the matched groups are shown as the SMD (Supplementary Figure 5), and P values were determined by logistic regression analysis (Table 4). As we expected, all SMD values were lower than 0.1, and the P values were higher than 0.05, suggesting that the data were balanced. Then, we performed K-M survival analysis and found that patients with SRC had poorer survival than those with MGC (P<0.0001), consistent with the CSS results of the two groups (P<0.0001) (Figure 6). Similarly, we performed PSM with our data by matching 45 MGC patients with 45 SRC patients (Table 5) and found that patients with SRC had poorer survival than those with MGC (P=0.03) (Figure 7). However, in the survival analysis for patients with early-stage GC, we found that the survival of patients with MGC was poorer than that of patients with SRC after matching (Supplementary Figure 6 and Figure 8).

**Table 4:** Characteristics of patients at diagnosis after PSM.

**Variables**	**Total (%)**	**Signet ring cell carcinoma**	**Mucinous Adenocarcinoma**	**P Value**
n	1484	742	742	
Age				0.051
<40	32(2.15%)	18(2.42%)	14(1.89%)	
<70	650(43.8%)	302(40.7%)	348(46.9%)	
≥70	802(54.04%)	422(56.87%)	380(51.21%)	
Race				0.09
White	1037(69.88%)	513(69.14%)	524(70.62%)	
Black	199(13.41%)	91(12.26%)	108(14.56%)	
Other	248(16.71%)	138(18.6%)	110(14.82%)	
Sex				0.503
Male	1014(68.33%)	501(67.52%)	513(69.13%)	
Female	470(31.68%)	241(32.47%)	229(30.86%)	
Lymph node Metastasis				0.097
NO	569(38.34%)	300(40.43%)	269(36.25%)	
Yes	915(61.66%)	442(59.57%)	473(63.75%)	
Metastasis				0.147
No	1198(80.73%)	588(39.62%)	610(82.21%)	
Yes	286(19.27%)	154(20.75%)	132(17.79%)	
Localization				0.059
Cardia	479(32.28%)	226(30.46%)	253(34.1%)	
Fundus	55(3.7%)	31(4.18%)	24(3.23%)	
Body	134(9.03%)	72(9.7%)	62(8.36%)	
Antrum	401(27.02%)	208(28.03%)	193(26.01%)	
Pylorus	45(3.03%)	26(3.5%)	19(2.56%)	
Lesser curvature	143(9.64%)	81(10.92%)	62(8.36%)	
Greater curvature	60(4.04%)	26(3.5%)	24(3.23%)	
Overlappping/NOS	167(11.25%)	72(9.7%)	105(14.15%)	
Size				0.268
≤2cm	168(11.32%)	87(20.55%)	81(10.77%)	
≤3cm	221(14.89%)	123(15.61%)	98(13.03%)	
≤5cm	424(28.57%)	205(24.98%)	219(29.12%)	
>5cm	671(45.22%)	327(38.86%)	344(47.07%)	
Regional_nodes_examined				0.479
0	267(18%)	138(18.6%)	129(17.39%)	
≤4	153(10.31%)	82(11.05%)	71(9.57%)	
>4	1054(71.02%)	512(69%)	542(73.04%)	
Historic Stage A				0.308
Localized	410(27.63%)	209(28.17%)	201(27.09%)	
Regional	702(47.44%)	338(45.56%)	366(49.32%)	
Distant	370(24.93%)	195(26.28%)	175(23.58%)	
T1 Stage				0.171
Tis	8(0.40%)	4(0.54%)	4(0.54%)	
T1	222(14.96%)	126(16.98%)	96(12.94%)	
T2	693(46.7%)	327(44.07%)	366(49.32%)	
T3	397(26.75%)	201(27.09%)	196(26.42%)	
T4	164(11.05%)	84(11.32%)	80(10.78%)	

**Table 5:** Basic characteristics of patients after PSM from the First Affiliated Hospital of Nanchang University.

**Variables**	**Total (%)**	**Signet ring cell carcinoma**	**Mucinous Adenocarcinoma**	**P Value**
n	90	45	45	
Age				0.694
<=45	7 (7.78%)	3 (6.67%)	4 (8.89%)	
>45	83 (92.22%)	42 (93.33%)	41 (91.11%)	
Sex				0.654
Male	60 (66.67%)	29 (64.44%)	31 (68.89%)	
Female	30 (33.33%)	16 (35.56%)	14 (31.11%)	
Lymph node Metastasis				0.286
N0	31 (34.44%)	17 (37.78%)	14 (31.1%)	
N1	21 (23.33%)	7 (15.56%)	14 (31.1%)	
N2	15 (16.67%)	7 (15.56%)	8 (17.78%)	
N3	23 (25.56%)	14 (31.1%)	9 (20%)	
Metastasis				0.999
No	86 (95.56%)	43 (95.56%)	43 (95.56%)	
Yes	4 (8.88%)	2 (4.44%)	2 (4.44%)	
T Stage				0.103
T1	15 (16.67%)	8 (17.78%)	7 (15.56%)	
T2	17 (18.89%)	4 (8.88%)	13 (28.89%)	
T3	11 (12.22%)	7 (15.56%)	4 (8.88%)	
T4	47 (52.22%)	26 (57.78%)	21 (46.67%)	
Localization				0.925
Cardia	4 (2.22%)	1 (2.22%)	1 (2.22%)	
Fundus	4 (2.22%)	1 (2.22%)	1 (2.22%)	
Body	18 (20%)	10 (22.22%)	8 (17.78%)	
Anturm	63 (70%)	32 (71.11%)	31 (68.89%)	
Overlappping/NOS	6 (6.67%)	2 (4.44%)	4 (8.89%)	
Size				0.821
≤2cm	17 (18.89%)	8 (17.78%)	9 (20%)	
≤3cm	18 (20%)	10 (22.22%)	8 (17.78%)	
≤5cm	38 (42.22%)	20 (44.44%)	18 (40%)	
>5cm	17 (18.89%)	7 (15.56%)	10 (22.22%)	
Examined_LNs				0.535
≤16	12 (13.33%)	7 (15.56%)	5 (11.11%)	
>16	78 (86.67%)	38 (84.44%)	40 (88.89%)	
Treatment methods				0.237
Conditional surgery	6 (6.67%)	5 (11.11%)	1 (2.22%)	
Laparoscopic surgery	78 (86.67%)	37 (82.22%)	41 (91.11%)	
Robotic surgery	6 (6.67%)	3 (6.67%)	3 (6.67%)	
Chemotherapy after surgery				0.824
No	59 (65.56%)	30 (66.67%)	29 (64.44%)	
Yes	31 (34.44%)	15 (33.33%)	16 (35.56%)	
Lymphatic vessel infiltration				0.832
No	42 (45.56%)	21 (46.67%)	20 (44.44%)	
Yes	49 (54.44%)	24 (53.33%)	25 (55.56%)	

## DISCUSSION

To the best of our knowledge, MGC is a rare subtype of GC, and few large-population studies have examined differences in the clinical features and prognosis of SRC and MGC. In our study, we included 6017 patients from the SEER database and comprehensively analyzed the differences in survival between SRC and MGC. Additionally, we extracted data for 266 patients from our hospital records to improve the reliability of the findings. Our study demonstrates that MGC does have a better prognosis than SRC via PSM and competing risk model analysis. However, in terms of early-stage disease, patients with MGC have a poorer prognosis than those with SRC.

MGC is rare and accounts for 3-10% of GC cases, while SRC accounts for 8-30% of GC cases [17–19]. Although their incidence rates are low, MGC and SRC are important tumor types because of their high malignancy and poor prognosis [11]. With respect to the clinical characteristics, SRC mainly occurs in younger patients, ranging from 50-60 years old. SRC appears to be more frequent in female patients and in Asian or other ethnic groups than MGC and is more frequently diagnosed in the early stage, which is in concordance with our study [18, 20]. In addition, the difference in prognosis between SRC and MGC is controversial. In line with our results, some studies have indicated that patients with SRC have a worse survival rate than those with MGC [21–23]. Some studies have suggested that patients with SRC have survival similar to those with MGC [24, 25]. In our study, we used PSM to adjust for confounding factors, increasing the robustness of the results [26]. In the group of patients from the SEER database, we found that SRC could be an independent risk factor for predicting survival after PSM. Moreover, considering the limitations of the information provided by the SEER database, we extracted patients from our hospital records and adjusted for other confounding factors, such as chemotherapy, lymphatic vessel invasion and treatment method; we found that SRC was a predictor of a poor prognosis. Furthermore, since being proposed in 1972, competing risk models have been regarded as highly suitable for the clinical prediction of prognosis because these models consider the existence of a competitive risk relationship with an observable end point [27]. In both Gray’s univariate and multivariate competing risk regression models, SRC was a risk factor compared to MGC. With regard to the possible causes, some studies have identified that SRC has a greater incidence of LNM, a greater risk of peritoneal metastasis, a greater risk of recurrence and lower chemosensitivity than MGC [23, 28], and these differences are closely associated with the different molecular characterizations of SRC and MGC [29].

Tsenga et al. conducted a larger population-based study with 2637 patients and found that patients with MGC were inclined to have more poorly differentiated tumors and greater tumor infiltration than those with SRC [4], which could explain our results regarding the survival difference among patients with early-stage GC [4]. Moreover, considering information from both the SEER database and our hospital, the results demonstrated that SRC was more frequently considered early-onset GC, found in females and diagnosed as early-stage GC, which may have contributed to the better survival outcomes of SRC than those of other histological types of GC. Recent studies have shown that patients with early-stage SRC have a lower risk of LNM, resulting in early-stage SRC having a favorable prognosis [30, 31]. Similarly, some studies have reported that patients with SRC have a significantly better survival rate than those with non-SRC [18, 24]. Potential factors related to the better survival include a younger age at diagnosis and a lower incidence of lymph node invasion in early-stage SRC patients [18]. However, some studies have suggested that there is no difference in the survival of early GC patients between SRC and other types of GC [20], while SRC was found to be a risk factor for predicting survival in patients with advanced cancer [19, 21]. The heterogeneous populations are an important reason for the inconsistent findings and differences in the results. With regard to the opposite survival trends of SRC in the early stage and late stage, some researchers have considered the SRC type of early GC to be characterized by a latent state with low aggressiveness and suggested that tumor invasiveness could significantly increase and accelerate when tumor cells invade the submuscular layer, resulting in a high risk of peritoneal metastasis [32]. Especially for those with CDH1 mutations, patients with SRC have poor chemosensitivity and a greater risk of metastasis [32, 33].

Finally, our study has some limitations that need to be discussed. First, we only focused on the OS and CSS of patients without considering cancer recurrence or disease-free survival, limiting our results in terms of clinical application. However, a competing risk model was developed to assess the value of histology in predicting survival by considering death unrelated to cancer. Second, the current analysis of the patient population could not exclude the possibility of selection bias. Therefore, interpretation of the survival differences between SRC and MGC requires caution.

In conclusion, our results indicate that compared to SRC, MGC is characterized by better survival. However, when considering early-stage GC, patients with MGC have a worse prognosis than those with SRC.

## MATERIALS AND METHODS

### Patients

The data of all patients with GC were retrieved from the SEER database with the National Cancer Institute’s SEER*Stat software (version 8.3.6). The patients did not provide informed consent because the SEER database is free for public use. All patients underwent surgery without chemotherapy. According to the International Classification of Diseases in Oncology (ICD-O-3), tumors with a code of 8490 were identified as SRC, while those with a code of 8481 or 8480 were considered MGC. In our study, patients were included according to the following criteria: (1) age of more than 20 years and diagnosis of GC by positive histology from 2004 through 2015; (2) histopathological type of SRC or MGC; (3) available survival information; and (4) available detailed information, including age, race, grade, number of regional lymph nodes (LNs) examined, tumor size, historic stage A, T stage, N stage and M stage. Detailed information on the excluded patients is listed in Supplementary Figure 1. In addition, we extracted data for 266 patients diagnosed with SRC or MGC from March 2014 to March 2020 at the First Affiliated Hospital of Nanchang University. All patients were followed up by telephone. Patients were included according to the following criteria: (1) age of more than 20 years and treatment with surgery; (2) diagnosis of SRC or MGC by pathology from March 2014 to March 2020; and (3) no serious chronic diseases, such as chronic renal failure. Patients were excluded according to the following criteria: (1) no record of TNM stage, tumor size, lymphatic vessel invasion or number of examined LNs; (2) chemotherapy before surgery; and (3) no information regarding survival. The study was approved by the Ethics Committee of the First Affiliated Hospital of Nanchang University. Detailed information is shown in Supplementary Figure 2.

### Clinicopathological factors

The patients extracted from the SEER database and our hospital for our study were divided into the SRC group and the MGC group. The patients from the SEER database were divided into two age groups: <=45 and >45 years; patients at our hospital were divided into two age groups: <=45 and >45 years. Race was classified into three types: white, black and other. Sex included male and female. Historic stage A was recorded as localized, regional or distant. T stage was recorded as T1, T2, T3 or T4. LN metastasis (LNM) was described as N0 (negative), N1 (1-2 positive LNs), N2 (3-6 positive LNs) or N3 (>6 positive LNs). M1 (Yes) indicated a positive M stage. Tumor size was categorized into 4 groups: ≤2 cm, ≤3 cm, ≤5 cm, and >5 cm. With respect to the number of examined LNs, the cut-off value was 16, according to previous studies [34]. The primary sites were recorded as the cardia, fundus, body, antrum, pylorus, lesser curvature, greater curvature and overlapping lesion/not otherwise specified (NOS). Similarly, the tumor sites in our patients were recorded as the cardia, fundus, body, antrum and overlapping lesion/NOS. All data of the patients from the SEER database are listed in Table 1, while the data of the patients from our hospital are listed in Table 2. The primary observation indicators were OS and CSS. CSS was defined as the duration from either the date of diagnosis or the start of treatment for cancer to the date of death from cancer.

### Statistical analysis

For basic statistical analysis, the patients were divided into two groups, namely, the SRC and MGC groups, and Pearson’s chi-squared test was utilized to investigate the associations among categorical variables. To explore the potential risk factors for CSS, we performed univariate and multivariate Cox regression, and the results are presented as the hazard ratio (HR) with 95% confidence interval (CI). With respect to the OS and CSS of patients with SRC and MGC, we created survival curves using R software. For the competing risk model, we constructed the model as described in a previous study [35]. Briefly, we selected CSS as the outcome of interest, whereas death due to other causes was considered a competing risk event, and a patient who was alive was regarded as a censored event. We created cumulative risk curves using Fine and Gray’s competing risk regression analysis. In addition, a multivariate competing risk model was used to explore the potential risk factors for CSS by R software.

Regarding the imbalance between the SRC and MGC groups, we performed PSM and inverse probability of treatment weighting (IPTW) to obtain new data for analysis with the MatchIt package in R software. The caliper value was set as 0.02, and the effect was evaluated based on the standardized mean difference (SMD) and P value. The effect was balanced when the SMD was less than 0.1 or the P value was greater than 0.05 [36]. The detailed process was as follows. First, we calculated the propensity scores of each patient according to histological type (SRC and MGC) with the multivariate logistic regression model. Then, we matched patients between the two groups at a ratio of 1:1. Next, we analyzed the differences in all variables between the SRC and MGC groups with the chi-squared test. Finally, we explored the correlation between survival and histological type by performing a K-M survival analysis.

All statistical analyses were performed with R software (version 3.6.1, StataCorp LLC, College Station, Texas). For comparisons among different patient groups, the chi-squared test was used for categorical variables, Student’s t-test was used for continuous variables with a Gaussian distribution, and the nonparametric Kruskal-Wallis rank-sum test was used for nonnormally distributed continuous variables or ordinal categorical variables. The chi-squared test was carried out with SPSS (version 24.0). The results were considered statistically significant when the P value was less than 0.05.

### Ethics statement

Ethics approval and consent was obtained from SEER database.

## Supplementary Material

Supplementary Figures
