# Supplementary material for: Prognostic analysis of gastric signet ring cell carcinoma and mucinous carcinoma: a propensity score-matched study and competing risk analysis
Source: Aging (Albany NY). 2020 Oct 31;12(21):22059–77. doi: 10.18632/aging.104048 (PMC7695374; doi:10.18632/aging.104048)
Supplement: Supplementary Figures [file aging-12-104048-s001..pdf]

SUPPLEMENTARY FIGURES

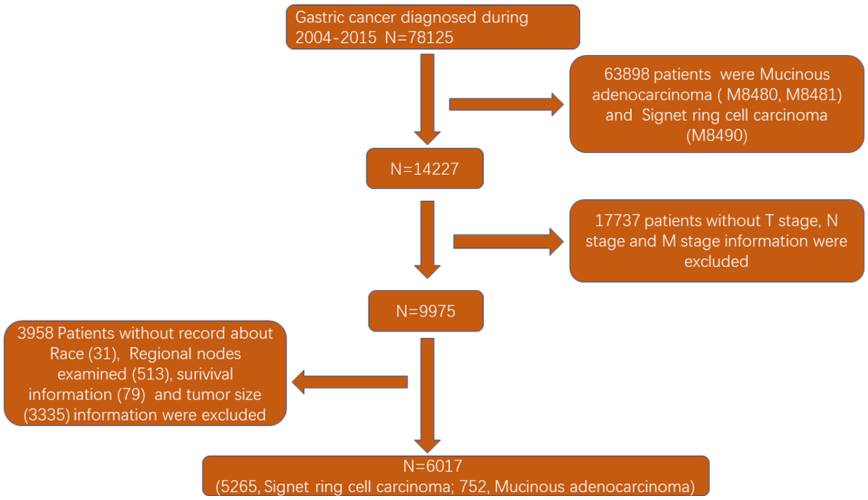

Supplementary Figure 1. Flowchart extraction of patient information from the SEER database in our study.

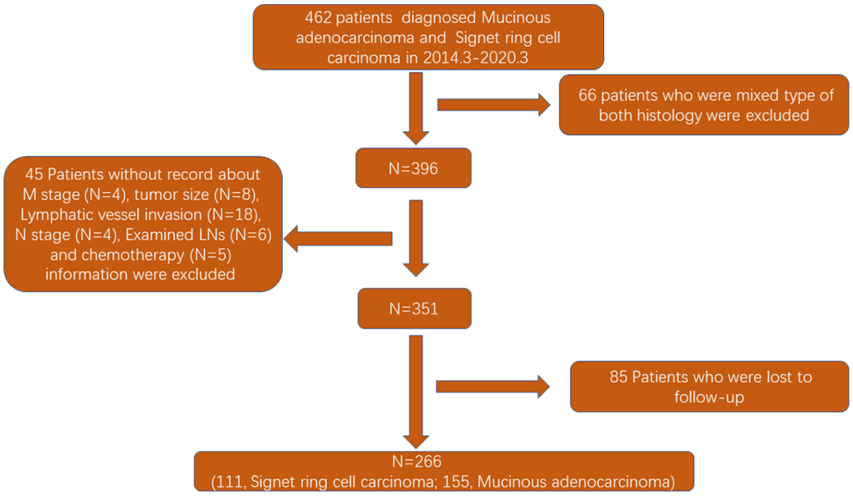

Supplementary Figure 2. Flowchart of extraction of patient information from the First Affiliated Hospital of Nanchang University in our study.

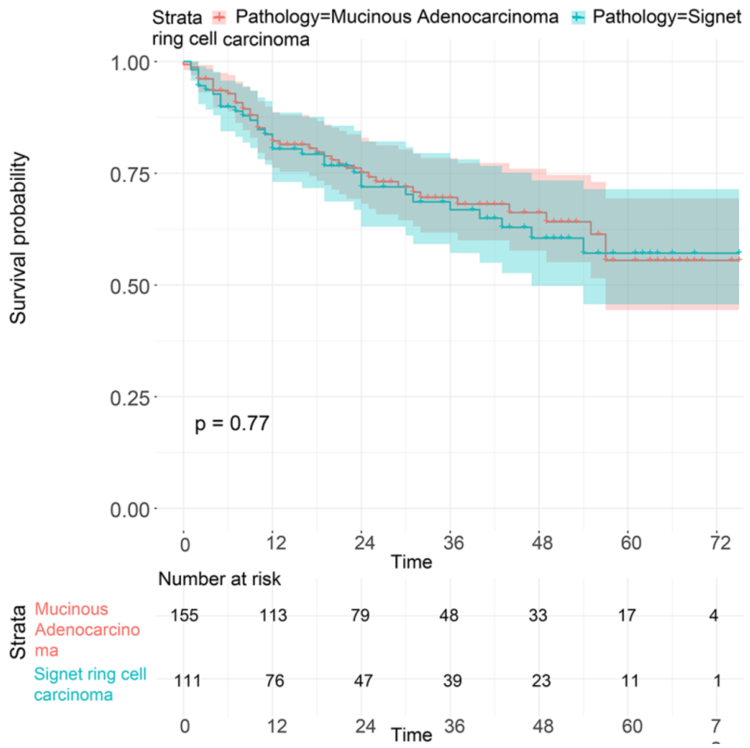

Supplementary Figure 3. Survival analysis of patients from the First Affiliated Hospital of Nanchang University.

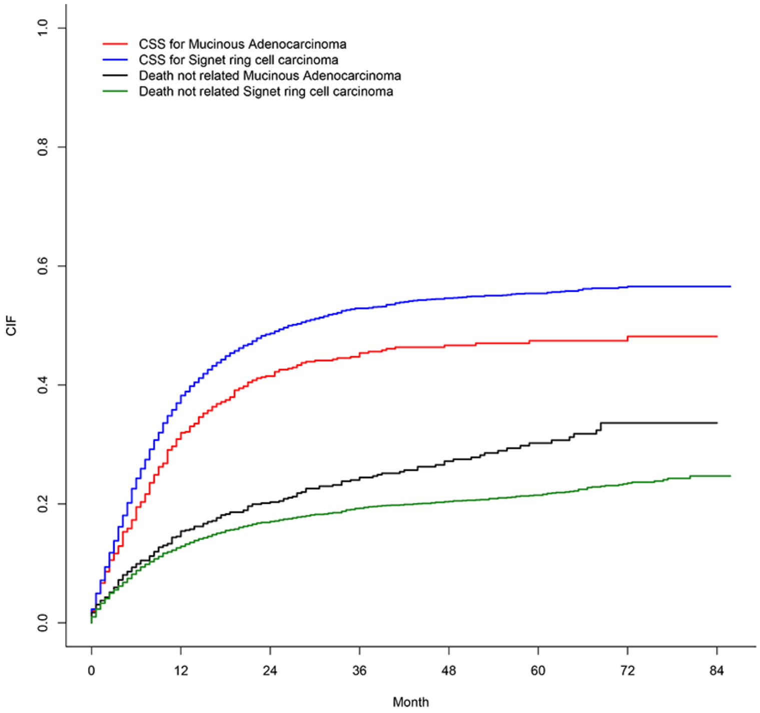

Supplementary Figure 4. Univariate analysis in the competing risk model to evaluate the value of histology (SRC and MGC) in predicting survival.

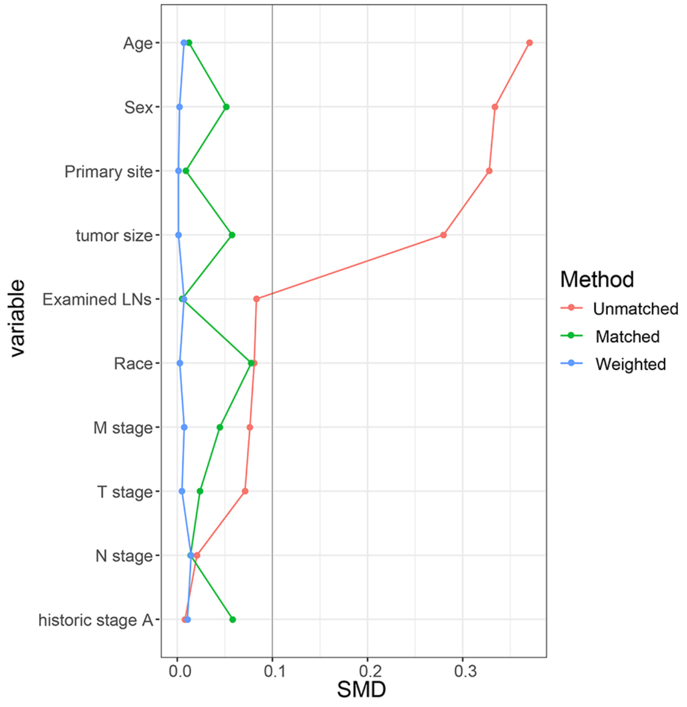

Supplementary Figure 5. SMD across covariates before and after PSM while exploring the association between survival and histology.

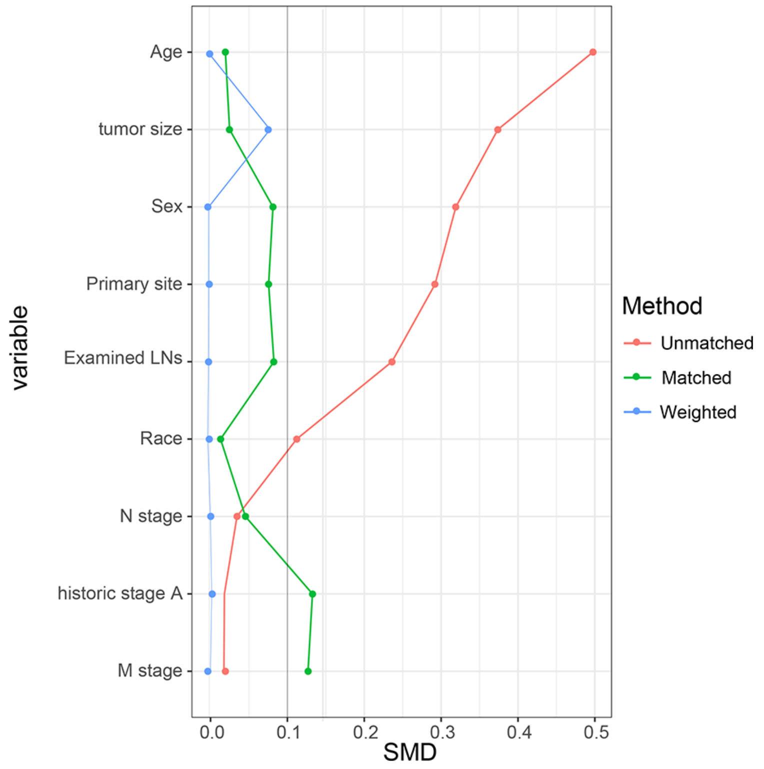

Supplementary Figure 6. SMD across covariates before and after PSM while exploring the association between survival and histology in early-stage GC.
